# Providing colored photoperiodic light stimulation during incubation: 2. Effects on early posthatch growth, immune response, and production performance in broiler chickens

**DOI:** 10.1016/j.psj.2021.101328

**Published:** 2021-06-12

**Authors:** Xujie Li, Bruce Rathgeber, Nancy McLean, Janice MacIsaac

**Affiliations:** ⁎ Department of Animal Science and Aquaculture, Faculty of Agriculture, Dalhousie University, Truro, NS, B2N 5E3, Canada; † Department of Plant, Food, and Environmental Sciences, Faculty of Agriculture, Dalhousie University, Truro, NS, B2N 5E3, Canada

**Keywords:** photostimulation, light color, incubation, thermoregulation, IgG

## Abstract

Previous findings have reported that providing light during incubation can affect hatchability and chick quality. This study was conducted to investigate the effects of providing light during incubation on posthatch broiler production parameters, thermoregulation and immune response. Lights with different wavelengths were used over the course of four separate hatches. Ross 308 broiler hatching eggs were randomly distributed into 4 lighting treatments for each hatch. The incubation lighting treatments included: dark as control, white, red, or blue lights for 12 h d^−1^ (200 lux at egg level). Broilers hatched from each incubator with the same gender were placed into one of 8 sets of pens (3 pens/set) and raised under 18 h d^−1^ photoperiod. Six birds per pen were immunized intraocularly with AviPro ND-IB Polybanco vaccine on d 10 and 21 posthatch. Chicks hatched under white and blue lights had heavier (*P* < 0.05) body weight and higher (*P* < 0.05) feed consumption than the control group during the first 6 h postplacement. No differences in vent temperature were found among treatments at 24 h posthatch (*P* > 0.05). Chicks hatched with light stimulation however had more stable (*P* < 0.05) cloaca temperature at 36 h posthatch. No differences in average body weight gain, feed consumption or feed conversion ratio were found among lighting treatments between d 7 and d 35. On d 14 of age, birds hatched from red light had higher (*P* < 0.05) total IgG concentration than those hatched under dark, blue or white light. These results indicated that in ovo light stimulation with different wavelengths did not affect growth parameters of broilers at market age. Providing photoperiodic blue and white light during incubation improved the production parameters of broilers during the first week posthatch.

## INTRODUCTION

Significant changes in genetics, nutrition and management have taken place in the broiler industry during the past decades. Broilers can achieve double their body weight in half the time when compared to broiler production in the 1950s (Havenstein et al., 2003). The National Chicken Council (2020) reported that the average slaughter age (at a weight of 2.86 kg) of a broiler is 47 d, which means the broiler spends almost one-third of its life as an embryo inside the incubator. Therefore, providing the optimum incubation environment is crucial for successful broiler production.

Light is an important exogenous environmental factor for controlling many physiological and behavioral processes in birds. It has been reported that avian species have a wider visual spectrum range than humans (Prescott and Wathes, 1999). Artificial lighting programs are a management tool for improving birds’ behavior, welfare, and production efficiency of commercial poultry (Rozenboim et al., 2004b; Olanrewaju et al., 2006; Schwean-Lardner et al., 2013; Sultana et al., 2013). Light intensity, the composition of the spectrum and photoperiod (daily pattern of light and dark exposure) are 3 parameters of light that need to be considered when used as tools to manage poultry production. Effects of providing different colors of light during rearing period on growth rates have been well documented. Broilers reared under blue or green light gained more weight than birds exposed to red or white light (Wabeck and Skoglund, 1974; Rozenboim et al., 1999; Rozenboim et al., 2004b). Soliman and Hassan (2019) reported that broilers reared under blue and white light gain significantly more weight and improve feed conversion ratio than birds reared under red light. The increased body weight might be due to increased satellite cell proliferation when exposed to light with a short wavelength (Halevy et al., 1998). Whether providing light illumination with blue light to chicken embryos had a similar effect on growth rate of broilers posthatch was investigated in this study.

The concept of providing illumination during incubation has been a subject for many years. As far back as the late 1960s, accelerated embryo development was found when White Rock eggs were stimulated with continuous light exposure (Siegel et al., 1969). Conventional light sources, fluorescent and incandescent bulbs, however, may produce additional heat and alter the incubation environment. Light emitting diode (**LED**) fixtures have become widely available as they feature durability, low heat production, and energy efficiency. LED bulbs are available in monochromatic colors and capable of being dimmed. Several studies have demonstrated that providing light with LED bulbs during incubation has positive effects on embryo development (Shafey and Al-mohsen, 2002; Rozenboim et al., 2004a; Wang et al., 2014), chick quality at hatch (Huth and Archer, 2015; Archer, 2017; Archer et al., 2017), and welfare posthatch (Huth and Archer, 2015; Archer and Mench, 2017). In ovo intermittent light illumination (15 min light, 15 min dark) during incubation increased the number of skeletal muscle cells and enhanced satellite cell proliferation in broilers on d 1 and 3 posthatch (Halevy et al., 2006). Their results suggested that light stimulation during incubation could affect myogenic activity by modulating energy metabolism and interact with hormones involved in growth control. Plasma growth hormone concentration has been found to reach peak levels at late embryonic and early posthatching development, and then gradually declined until close to sexual maturation (Scanes et al., 1992). Light spectrum has varying effects on humoral and cellular immune responses on broilers during rearing period. For example, T-lymphocyte (Xie et al., 2008b) and splenocyte (Xie et al., 2008a) proliferation were the highest in broilers exposed to white light through the entire experimental period. However, broilers reared under green light had higher anti-Newcastle disease virus (**NDV**) antibody production than those under white light at d 42 of age (Xie et al., 2008b). Higher anti-NDV antibody titer was found in broilers under green and blue light than those under white and red light for the entire production period (Zhang et al., 2014). However, Firouzi et al. (2014) reported that green light only promotes anti-NDV antibody production at the early growth stage, compared to red light. No differences in humoral response were found among light color treatments on d 30 and 42. These findings suggest that light source, broiler strain, vaccination and sampling day may account for the variation in the effects of light wavelength on immune response. Those studies were focused on the effects of light illumination during rearing period. The study conducted by Archer and Mench (2013) found that broilers incubated with a light program of 12L:12D had a stronger humoral immune response to keyhole limpet hemocyanin, which is a nonpathogenic protein antigen and has often been used to evaluate humoral immune response. The information regarding the effects of providing light with different spectra during incubation on posthatch immune response on broilers is limited and will be tested in the current study.

A bird's development during brooding stage is important for their future performance. After transferring to the grow-out facility, the changes in nutritional resources and environmental factors (temperature, humidity, light exposure, and handling) may be challenging for young chicks. Provision of a photoperiod during incubation may entrain the circadian rhythms of melatonin secretion in broiler embryos and provide positive effects on the endocrine, neuronal and immune systems, and improve behavioral processes at a young age. Optimal light regimes during incubation need to be thoroughly investigated as a potential environmental tool to improve young chicks’ adaptation to the posthatch environment.

The objective of this study was to evaluate the effects of providing a photoperiod light during incubation on early growth performance, production parameters and immune response in broilers. We hypothesized that providing blue and white LED lights for 12 h d^−1^ during incubation have positive effects on body weight gain, food intake, and thermoregulation of newly hatched chicks during an early age and humoral immune response to Newcastle-bronchitis vaccine in Ross 308 broilers.

## MATERIALS AND METHODS

The experimental protocol was carried out in according with the Canadian Council of Animal Care Guideline (CCAC, 2009). Ross 308 hatching eggs were incubated under white (color temperature 4100K, Canarm, Brockville, ON, Canada), red (Once Innovations, Plymouth, MN), or blue (Once Innovations, Plymouth, MN) LED lights for 12 h d^−1^ at 200 lux for the entire incubation period, and the dark incubation condition served as control. The study was conducted in four repeated hatch trials (n = 2,176, 1,664, 1,696 and 1,600 eggs for four trials, respectively). In each trial, eggs were randomly assigned to four lighting treatments with 2 replicate single-stage incubators (ChickMaster G09, Cresskill, NJ). The details of the incubation treatments and hatching performance are given in Li et al. (2021)

### Animals and Husbandry

After hatch, broilers were placed into 48 pens with all chicks within a pen from the same incubator. There were 25 ± 1 birds of the same lighting treatment and gender in each pen. The birds were raised under an 18 h d^−1^ photoperiod (0700 to 0100 h, which turned on at the same time as during the incubation period) with a photophase light intensity of 20 lux (Philips F17T8/TL835 17-W fluorescent tubes, color temperature at 3500K) during the first 4 d and gradually decreased to 5 lux on d 9 of age. The brooding temperature was 30 to 32°C during the first 6 d. Each pen (2.19 m × 1.00 m) was prepared with new, clean, wood shavings at a depth of 4 cm. The stocking density was 0.08 m^2^ bird^−1^. Groups of 3 pens with the same treatment combination (incubation lighting × gender) from the same incubator were used as an experimental unit for the posthatch performance portion of the research. The diets were formulated to meet or exceed National Research Council (1994) nutrient requirements. All birds were fed the same diets ad libitum within 3 growth phases. The nutritionally balanced starter diet in crumble form was supplied from d 0 to 14. The grower and finisher diets in pellet form were supplied from d 15 to 25 and d 26 to 35, respectively. The starter diet was provided in a plastic 50 cm trough feeder (Little Giant, Miller Manufacturing, Eagan, MN) during the first 5 d after placement. After d 5 of age, feed was provided from tube feeders. Water was provided ad libitum from 2 nipple drinkers per pen.

### Broiler Growth Performance

**Figure 1:**
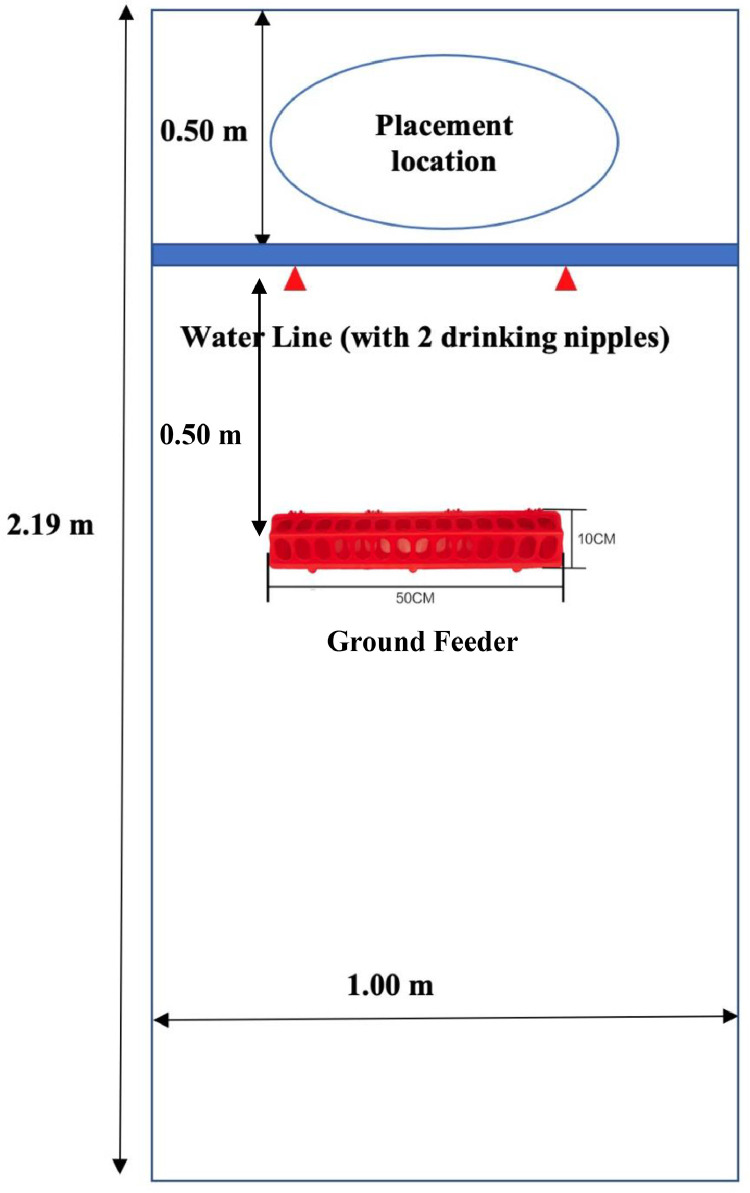
Floor pen dimension and chick placement location.

Following completion of chick processing at the hatchery (at 1 pm), chicks were group weighed and placed in stages with a 5 min interval between pens. Each round of placement consisted of all 8 treatment combinations to minimize the effects of placement time. Chicks were placed on the litter behind the water line (Figure 1) without guiding them to find feed and water. At exactly 6 h postplacement, body weight and the remaining feed were weighed in the same order as placement to calculate the feed consumption and body weight gain during the first 6 h of access to feed and water. Chicks in each pen were weighed every 24 h and feed consumption was measured daily during the first 7 d. The birds were group weighed on d 14, 25, and 35. The feed remaining in the feeders was weighed on each weigh day and as mortality occurred. Mortality was recorded and sent to a veterinary pathologist for necropsy (Animal Health Laboratory, Truro, NS, Canada). Growth performance was evaluated using feed consumption, body weight, body weight gain, and feed conversion ratio (**FCR**).

### Body Temperature

Cloaca temperature of 5 randomly selected chicks per pen was measured with a digital thermometer (Braun IRT-4020, Braun, Kronberg, Germany) at 24 h posthatch (8 am). This process was repeated 12 h later (at 8 pm).

### Vaccination, Organ and Blood Sampling

Six birds per pen received a AviPro Newcastle-Bronchitis vaccine (B1 Type, B1 Strain, Mass. and Conn. Types, Live Virus, Elanco, Guelph, ON, Canada) via topical ocular at one drop/dose/chicken on d 10 and a booster vaccine was applied on d 21 in the second, third and fourth trials, according to the manufacturer's instructions.

One vaccinated chicken per pen (3 birds per experimental unit) was weighed individually at d 10, 14, 21, 25 and 35, and euthanized with an electric stunning knife. Blood samples were collected from the jugular vein immediately after euthanasia. Approximately 5 mL of blood was collected into nonheparinized tubes, incubated at room temperature for 4 h, and centrifuged at 2,000 x g for 10 min. The serum was separated and stored at -80°C until further analysis. The liver, spleen and bursa of Fabricius were excised and weighed and expressed on a relative body weight basis (g kg^−1^). A drop of blood collected from birds at d 35 of age was smeared on a glass slide. The smears were fixed and stained using Hema-3 (Fisher Science). One hundred leukocytes per slide were counted twice and the heterophil to lymphocyte (**H/L**) ratio was calculated.

### Serum Immunoglobulin

The total IgG concentration in serum was measured by using chicken IgG sandwich enzyme-linked immunosorbent assay (**ELISA**) quantitation and Starter Accessory Kits (Bethyl Laboratories, Montgomery, TX) following the manufacturer's procedure. The plates were read using a microplate reader (Bio-Tek Instrument Inc., Winooski, VT) at 450 nm. The four-parameter logistic model was found to be the best model to describe the IgG concentration and absorbance readings. The concentration of serum IgG was calculated. The inter- and intra-assay % CV were both under 5%.

### Statistical Analysis

The experiment was a randomized complete block design with a set of 4 incubators as one block (2 blocks per trial). Three pens of birds hatched from the same incubator per trial were used as the experimental unit with 8 blocks for each treatment combination (lighting × gender). One incubator (dark-control) in trial 2 was not functioning as consistently as the other 7 incubators and was removed from the analysis. Growth performance during the first 6 h, vent temperature on d 1 of age, organ development, H/L ratio, and serum IgG concentration were analyzed using the Mixed Procedure of the SAS v. 9.4 (SAS Inc., Cary, NC, 2013). All broiler body weight, feed consumption, body weight gain and feed conversion ratio were analyzed as repeated measures by using the Mixed Procedure of SAS v. 9.4 (SAS Inc., Cary, NC, 2013). In repeated measure analysis, four covariance structures, compound symmetry, variance components, first order autoregressive and unstructured covariance, which provided the smallest corrected Akaike Information Criterion and Bayesian Information Criterion absolute values, were selected to conduct the ANOVA test. The residuals of error met the assumptions of normal distribution, independently distribution with mean zero and had constant variance. In all cases, if significant effects were found, the Tukey-Kramer test was applied to differentiate the means at 5% level of significance.

## RESULTS AND DISCUSSION

### Broiler Growth Performance During an Early Age

Chick body weight at the time of placement and FCR during the first 6 h were not affected (*P* > 0.05) by the lighting treatment, gender or their combinations. Body weight ranged from 44.3 to 44.7 ± 0.21 g bird^−1^ at placement. However, the body weight following 6 h of accessing feed and water was affected (*P* < 0.05) by the incubation lighting treatment (Table 1). Chicks hatched from white and blue light illumination during incubation had significantly higher body weight at 6 h than those hatched under dark, and red light group was intermediate. Body weight gain during the first 6 h postplacement was affected (*P* < 0.05) by lighting treatments and gender (Table 1). Chicks hatched under white light with 12 h d^−1^ had significantly higher body weight gain than those hatched under dark. Females had higher (*P* < 0.05) body weight gain than males. Furthermore, higher (*P* < 0.05) feed intake was found during the first 6 h in white and blue groups than control (Table 1).

**Table 1:** Effects of providing different colored LED lights during incubation on body weight (g bird^−1^) at 6 h postplacement, body weight gain (g bird^−1^) (% of placement body weight) and feed consumption (g bird^−1^) during the first 6 h postplacement in broilers.

		Body weight at 6 h post placement			Body weight gain during the first 6 h postplacement			Feed consumption during the first 6 h postplacement		
Light	n^1^	Male	Female	Average	Male	Female	Average	Male	Female	Average
Dark	7	49.0 ± 0.30	49.0 ± 0.30	49.0 ± 0.22^b^	4.0 ± 0.36 (8.88%)	4.6 ± 0.36 (10.41%)	4.3 ± 0.26 (9.64%)^b^	3.2 ± 0.16	3.4 ± 0.15	3.3 ± 0.11^b^
White	8	50.0 ± 0.28	50.0 ± 0.28	50.0 ± 0.20^a^	5.1 ± 0.34 (11.38%)	5.4 ± 0.34 (12.18%)	5.2 ± 0.24 (11.78%)^a^	3.8 ± 0.14	3.8 ± 0.15	3.8 ± 0.10^a^
Red	8	49.4 ± 0.28	49.5 ± 0.28	49.4 ± 0.20^ab^	4.9 ± 0.34 (11.12%)	5.4 ± 0.34 (12.31%)	5.1 ± 0.24 (11.71%)^ab^	3.5 ± 0.15	3.7 ± 0.14	3.6 ± 0.10^ab^
Blue	8	49.4 ± 0.28	50.3 ± 0.28	49.9 ± 0.20^a^	4.7 ± 0.34 (10.56%)	5.6 ± 0.34 (12.73%)	5.2 ± 0.24 (11.65%)^ab^	3.7 ± 0.15	3.9 ± 0.14	3.8 ± 0.10^a^

Average		49.4 ± 0.14	49.7 ± 0.14		4.6 ± 0.17 (10.48%)^y^	5.2 ± 0.17 (11.91%)^x^		3.6 ± 0.07	3.7 ± 0.07	

Effect		ANOVA P-value								

Lighting (L)		0.006			0.039			0.007		
Gender (G)		0.164			0.017			0.257		
L x G		0.313			0.768			0.883		
Block		<0.0001			<0.0001			<0.0001		

^a,b^Means within a column with different letters differ significantly according to Tukey-Kramer test (α = 0.05).

^x,y^Means within a row with different letters differ significantly according to Tukey-Kramer test (α = 0.05).

Providing photoperiodic white lighting during incubation positively affected the chick growth during the initial part of the growing period in the current study. The feeding and exploratory behavior might be affected by the entrained circadian rhythms during incubation and lead to higher feed intake and body weight gain. Archer et al. (2009) reported that chicks hatched under dark had lower feed intake than those hatched with light exposure. The physiology and behavior of newly hatched chicks change rapidly from hour to hour during the postincubation period (Balážová and Baranyiová, 2010). Balážová and Baranyiová (2010) reported that male chicks had higher visual orientation behavior, while females had higher horizontal locomotor activity at d 3 of age during the first 10 min exposure to a novel environment. Our study also found that during the first 6 h in the grow-out pen, female chicks had higher body weight gain than males, which is considered increased exploratory behavior related to learning to eat. In order to test the hypothesis of behavior changes from incubation lighting treatment, an open-field behavior test would be useful to examine the response of newly hatched chicks to a novel environment in a future study. The mechanism of light illumination during incubation improving growth rate in neonatal chicks is still unclear. The higher body weight gain could be due to a higher amplitude of growth hormone secretion, which can be influenced by melatonin. There is evidence indicating melatonin influences growth by modulating growth hormone synthesis posthatch in avian species. A positive correlation between plasma melatonin concentration and growth hormone has been reported for broilers exposed to monochromatic light (Zhang et al., 2016). Exogenous melatonin administration increased plasma growth hormone concentration in Japanese quail (Zeman et al., 1999), mature pigeon (McKeown et al., 1975), and turkey (Fehrer et al., 1985). The development and maturation of photoreceptors starts during incubation. The enzymes involved in melatonin synthesis become functional during incubation and reach a peak before hatch (Espinar et al., 1994). Providing photoperiod for the whole incubation period resulted in a higher blood melatonin level in broiler chickens than those incubated under dark when placed in an environment with continuous light (Özkan et al., 2012). It is also possible that providing a photoperiod during incubation can lead to cerebral lateralization of visual pathways and alter the posthatch feeding behavior (Roger, 2008). Archer and Mench (2014a) found the melatonin rhythm on d 19 of incubation had a carry-over effect on the rhythmicity of general activity over a 6-wk growth period in broilers. Furthermore, increased plasma growth hormone and prolactin levels in broiler embryos have been found with in ovo green light exposure (Dishon et al., 2017). But it is important to note that the light regime applied during incubation in their study was 15 min light/15 min dark to avoid overheating of the eggs. A short duration of light exposure (3 min) during the scotophase decreased melatonin production by inhibiting NAT activity in rats (Klein and Weller, 1972). For avian species, as little as 5 min light exposure during the dark phase decreased NAT activity in both the retina and pineal gland and the suppressive effect of light exposure was spectrum related (Zawilska et al., 1995). Zawilska et al. (1995) reported that NAT activity in the pineal gland decreased by approximately 20% when exposure to white or green light for 5 min and NAT activity started to increase after 15 min of returning to darkness and reached the control level by 1 h for red light and 2 h for blue and green light. Pineal NAT activity was still lower than the control level after 2 h when chicks were exposed to white light for 5 min. Our study found that chicks hatched under white light had higher body weight gain at 6 h than those hatched under dark, which supports the hypothesis that providing photoperiodic blue light during incubation would positively affect body weight gain and feed consumption for young chicks.

Body weight was measured every 24 h during the first week to investigate the effects of photoperiodic light during incubation on growth performance during the brooding stage. Average body weight during the first week was significantly affected (*P* < 0.05) by the incubation lighting treatments (Table 2). No differences (*P* > 0.05) in body weight gain, feed intake or FCR were found among lighting treatments or their combination with other factors (gender or day). The white and blue groups had higher (*P* < 0.05) body weight than those incubated under dark (Table 3). Chicks in red group had an intermediate average body weight during the first week as we found the same trend at 6 h postplacement. Continuous green light stimulation during incubation has been shown to accelerate broiler weight gain and pectoral muscle weight by d 6 of age without improving feed intake (*P*-value: 0.08) and FCR (*P*-value: 0.93) (Zhang et al., 2016). It is possible that increased weight gain was associated with enhancing proliferation of skeletal muscle satellite cells by both green and blue light stimulation (Halevy et al., 1998). In our case, providing an in ovo photoperiod with shorter wavelength (blue light) may provide stronger stimulation to the photoreceptor cells than red light and dark, and entrained the rhythmic expression of core clock genes. Di Rosa et al. (2015) found that zebrafish larvae illuminated with blue light had higher *clock1* and *per1b* expression during the scotophase than those under white, red, and dark on d 7 postfertilization. The circadian rhythmicity is the output of rhythmic expression of core clock genes by positive and negative transcriptional feedback (Reppert and Weaver, 2002). The circadian rhythm of pineal melatonin synthesis can provide the internal temporal cue to the target tissue and regulate the production of somatotropic axis hormones (growth hormone and insulin-like growth factor-I) and the expression of growth hormone receptors (Dishon et al., 2017).

**Table 2:** ANOVA table of the effects of providing colored photoperiodic lighting treatments during incubation on first week growth performance in broiler chickens.

Effect	Body weight	Body weight gain	Feed consumption	FCR
Light (L)	0.001	0.7691	0.1496	0.7946
Gender (G)	0.487	0.9139	0.0069	0.0522
L x G	0.164	0.9340	0.5440	0.9402
Day (D)	<.0001	<.0001	<.0001	<.0001
L x D	1.000	0.9999	0.7404	0.9462
G x D	0.430	0.4481	0.3494	0.2466
L x G x D	1.000	1.0000	0.9998	0.9994
Block	<.0001	<.0001	<.0001	<.0001
Covariance structure	Variance components with square root transformation	First order autoregressive	First order autoregressive with log 10 transformation	First order autoregressive with log 10 transformation

**Table 3:** Effects of providing different colored LED lights during incubation on average body weight (g bird^−1^) during the first week in broiler chicks.

Light	n^1^	Male	Female	Average
Dark	7	101.8 ± 0.47	101.2 ± 0.47	101.5 ± 0.34^c^
White	8	103.1 ± 0.44	102.9 ± 0.44	103.0 ± 0.31^a^
Red	8	101.7 ± 0.44	101.9 ± 0.44	101.8 ± 0.31^bc^
Blue	8	101.9 ± 0.44	103.0 ± 0.44	102.5 ± 0.31^ab^

Average		102.1 ± 0.22	102.2 ± 0.22	

1 Number of experimental units. Experimental unit = 3 pens of birds hatched from the same incubator. ^a,b,c^Means within a column with different letters differ significantly according to Tukey-Kramer test (α = 0.05).

The source, spectrum, intensity and photoperiod are artificial light characteristics influencing modern poultry management. The effects of light spectra on chicken production, health and welfare have been well documented (Rozenboim et al., 2004b; Xie et al., 2008a,b; Sultana et al., 2013; Li et al., 2015; Dishon et al., 2017). Broilers reared under blue light, green light or their combination had higher body weight than those under white or red light (Rozenboim et al., 1999, 2004b). Similar effects were reported for turkeys (Rozenboim et al., 2003). In broiler production, constant or near-constant light was commonly applied during an early age to maximize the visual access to feed and water. Early studies showed a reduction in feed consumption and body weight gain immediately after introduction of a dark period (Classen and Riddell, 1989). However, the impact of introducing darkness on growth rate decreased with a longer introduction time. Riddell and Classen (1992) found that male broiler chickens raised under continuous light had higher body weight gain than those with photoperiods before d 42, especially during the first 21 d. However, at d 63 of age, the birds reared under increasing lighting treatment, which gradually increases the light period from d 7 to d 42, were heavier than those reared under continuous light. Rozenboim et al. (1999) also found a higher body weight gain after d 49 of age when broilers were reared under an increasing light schedule (23L:1D from d 1 to d 4, 8L:16D from d 5 to 14, then gradually increased to 16L: 8D by d 48). These results suggested that an increased duration of darkness resulted in lower initial body weight gain, followed by a faster compensatory growth later in the grow-out period. In addition, embryonic light stimulation has been shown to result in decreased stress level with lower corticosterone response to crating stressor (Archer and Mench, 2014b). Our study showed that providing white and blue photoperiodic light during incubation can effectively introduce the day-night cycle even before hatch and minimize the effect of introducing darkness posthatch, as improved chick growth was found during the first week.

### Overall Growth Performance

Providing white, blue or red light for 12 h per day during incubation did not affect (*P* > 0.05) body weight, body weight gain, feed consumption or FCR after one week of age (Table 4). In the current study, the chicks hatched under lighting treatments lost their weight advantage after d 7 of age (Supplementary Table 1), which may be due to the presence of grow-out photoperiod modulating rhythms of somatotropic axis hormones. Many studies have investigated the effects of providing light during incubation on posthatch growth performance, but the effects remain inconclusive. Some studies reported that light stimulation during incubation did not affect posthatch production parameters in broilers (Archer et al., 2009; Archer and Mench, 2014a; Huth and Archer, 2015; Archer, 2017) and Japanese quail (Sabuncuoglu et al., 2018; Hanafy and Hegab, 2019). In contrast to our results, improved body weight at market age by incubation with light was found in broilers (Zhang et al., 2016; Van der Pol et al., 2017) and turkey hens (Rozenboim et al., 2003). Interesting results were found by Zhange et al. (2012), who reported that continuous green light stimulation during incubation increased the body weight and feed intake posthatch, as compared to dark and blue light. The enhanced production parameters may be associated with increased number of satellite cells and proliferation and differentiation of myoblasts. The stimulatory effect on posthatch muscle growth depends on light spectrum, but not depends on photoperiod (Halevy et al., 2006). In addition, the transmittance of light through the shell may be influenced by light characteristics, spectrum and intensity, as well as characteristics of the eggshell, thickness, and pigmentation. Further studies are needed to investigate the light characteristics of different wavelengths penetrating through the eggshell and received by chicken embryo and whether blue light and green light are involved differently in regulation of growth hormone secretion in broilers. The combination of embryonic and posthatch lighting regimen would be a valuable topic to investigate the long term effects of light components on bird health, behavior, and growth performance.

**Table 4:** ANOVA table of the effects of providing colored photoperiodic lighting treatments during incubation on growth performance of broiler chickens during a 35-d period in the 2nd, 3rd, and 4th trials.

Effect	Body weight	Body weight gain	Feed consumption	FCR
Lighting (L)	0.3082	0.4891	0.4676	0.7518
Gender (G)	<.0001	<.0001	<.0001	<.0001
L x G	0.9878	0.9579	0.9972	0.9658
Day (D)	<.0001	<.0001	<.0001	<.0001
L x D	0.9983	0.9951	0.9619	0.9700
G x D	<.0001	<.0001	<.0001	0.2820
L x G x D	0.9995	0.7645	0.7267	0.8160
Block	<.0001	<.0001	0.0001	<.0001
Covariance structure	Variance components with log 10 transformation	First order autoregressive with square root transformation	First order autoregressive with square root transformation	Variance components

Total mortality from d 1 until d 35 was 2.55%, and it did not differ (*P* > 0.05) among incubation lighting treatments and did not interact with gender.

#### Body Temperature

An important finding from our study was the cloaca temperature at 36 h posthatch was affected by provision of photoperiodic lighting during incubation. No differences in cloaca temperature were found among treatments at 24 h posthatch (Table 5). However, the cloaca temperature at 36 h posthatch was lower (*P* < 0.05) in chicks hatched under dark than those hatched under white, red or blue light (Table 6). Birds maintained a relatively stable core body temperature via regulating a variety of thermoregulatory responses to support the balance between heat production and heat loss (Yahav, 2015). Newly hatched birds may require more than 3 d for endothermic thermoregulation to be fully functional (Wiebe and Evans, 1994). The ability to maintain a stable body temperature at young age may be due to an advanced embryo development and had a better ability to balance heat production and loss. Increased feed intake has been found to be associated with higher heat production (Zhou and Yamamoto, 1997). Heat conserving behavior may also account for the lower feed intake in chicks hatched under dark as being clustered to avoid heat loss may reduce the frequency of feeding behavior. The feeding behavior of neonatal chicks can be regulated by the daily rhythmic expression of orexigenic and anorexigenic neuropeptides in the hypothalamus (Furuse et al., 2001; Kaiya et al., 2009; Mishra et al., 2016). In addition, melatonin synthesis and secretion can regulate thermoregulatory mechanisms and energy metabolism in birds (Saarela and Heldmaier, 1987; Zhou and Yamamoto, 1997; Murakami et al., 2001; Isobe et al., 2002). Underwood and Edmonds (1995) reported that body temperature and activity were synchronized by cyclic administration of melatonin via drinking water in Japanese quail. The entrained circadian rhythm in body temperature was maintained up to 12 d. In ovo light stimulation changes the rhythmic secretion of melatonin and it may have an effect on thermoregulation in the bird after hatch. Our findings relate to the results obtained by Sinkalu et al. (2015) that broilers treated with exogenous melatonin via drinking water had lower and less fluctuating cloaca temperatures than those reared under continuous light from d 14 to 42 of age. The smaller differences in cloaca temperature between 24 and 36 h postplacement in birds hatched under photoperiodic light illumination indicated that lighting treatments during incubation may have positive effects on thermoregulation of neonatal chicks. Maintenance of a relatively stable core temperature provides a protective internal environment for efficient functioning of the cells, tissue, and organs (Nakamura, 2011). Although we did not measure the body temperature continuously during the brooding stage, considering the higher feed intake after placement and improved growth performance, we speculated that providing photoperiodic light stimulation during incubation increased the metabolic rate and heat production in broiler chicks during the photophase at a young age.

**Table 5:** Effects of providing different colored LED lights during incubation on cloaca temperature (°C) at 24 h posthatch in broiler chickens.

Light	n^1^	Male	Female	Average
Dark	7	39.9 ± 0.03	40.0 ± 0.03	39.9 ± 0.02
White	8	39.9 ± 0.03	40.1 ± 0.03	40.0 ± 0.02
Red	8	39.9 ± 0.03	40.0 ± 0.03	39.9 ± 0.02
Blue	8	39.9 ± 0.03	40.1 ± 0.03	40.0 ± 0.02

Average		39.9 ± 0.02^b^	40.1 ± 0.02^a^	

Effect		ANOVA *P*-value		

Lighting (L)		0.180		
Gender (G)		<0.0001		
L x G		0.963		
Block		<0.0001		

1 Number of experimental units. Experimental unit = 3 pens of birds hatched from the same incubator. ^a,b^Means within a row with different letters differ significantly according to Tukey-Kramer test (α = 0.05).

**Table 6:** Effects of providing different colored LED lights during incubation on cloaca temperature (°C) at 36 h posthatch in broiler chickens.

Light	n^1^	Male	Female	Average
Dark	7	39.7 ± 0.04	39.8 ± 0.04	39.7 ± 0.03^b^
White	8	39.8 ± 0.04	40.0 ± 0.04	39.9 ± 0.03^a^
Red	8	39.8 ± 0.04	40.0 ± 0.04	39.9 ± 0.03^a^
Blue	8	39.9 ± 0.04	39.9 ± 0.04	39.9 ± 0.03^a^

Average		39.8 ± 0.02^y^	39.9 ± 0.02^x^	

Effect		ANOVA *P*-value		

Lighting (L)		0.001		
Gender (G)		<0.0001		
L x G		0.171		
Block		<0.0001		

1 Number of experimental units. Experimental unit = 3 pens of birds hatched from the same incubator. ^a,b^Means within a column with different letters differ significantly according to Tukey-Kramer test (α = 0.05). ^x,y^Means within a row with different letters differ significantly according to Tukey-Kramer test (α = 0.05).

To the best of our knowledge, this is the first report on evaluating the effects of photoperiodic light stimulation during incubation on growth performance at placement, body temperature, and daily chick growth in broilers during the first week of production. The positive effects on chick growth demonstrated that providing photoperiodic blue and white light illumination during incubation can improve the adaptation of newly hatched chicks to grow-out environment. The effects were not found after d 7 of age, which may be a result of establishment of new circadian rhythm, which is entrained by the longer photoperiod during grow-out period.

#### Organ Weight, H/L Ratio, and Total IgG

Relative spleen weight was only affected (*P* < 0.05) by incubation lighting treatments on d 10 of age (Table 7). No differences (*P* > 0.05) in relative spleen weight were found among dark and light color treatments. We only found that birds hatched under red light had heavier (*P* < 0.05) relative spleen weight than those hatched under blue light (Table 8). Most photostimulation studies on immune function have focused on photoperiod or day length. Generally, a longer dark period increased the immune response and it was associated with increased melatonin secretion. Kliger et al. (2000) found increased splenic lymphocyte proliferation when broilers were given an intermittent lighting schedule compared to constant light exposure posthatch. We did not find an accelerated spleen development as measured by weight between dark and incubation lighting treatments. The relative weight of spleen and bursa of Fabricius can be affected by environmental factors, such as light (Li et al., 2015) and temperatures (Quineteiro-Filho et al., 2010). Lighter relative spleen and bursa of Fabricius were found in broilers exposed to a heat stress environment (Pardue et al., 1985). We hypothesized that birds hatched under red light may have higher cell proliferation in spleen than stimulated with short wavelength such as blue light according to the relative spleen weight on d 10. Heterophil to lymphocyte ratio (H/L) is used as an index to evaluate levels of stress in poultry. This ratio can increase during stressful situations, including environmental changes such as social stress (Gross and Siegel, 1983) or even lighting programs (Huth and Archer, 2015). A higher H/L ratio in blood was found when birds were raised under constant light compared to 12 h d^−1^ light treatment (Zulkifli et al., 1998). No differences in H/L ratio (*P* > 0.05) among incubation lighting treatments in the current study suggest that providing light illumination up to 12 h d^−1^ did not induce stress level during rearing period.

**Table 7:** ANOVA table of the effects of providing colored photoperiodic lighting treatments during incubation on relative organ weight of broiler chickens during a 35-d period in the 2nd, 3rd, and 4th trials.

	D 10			D 14			D 21			D 25			D 35		
Effect	Spleen	Bursa	Liver	Spleen	Bursa	Liver	Spleen	Bursa	Liver	Spleen	Bursa	Liver	Spleen	Bursa	Liver
Lighting (L)	0.038	0.388	0.566	0.901	0.817	0.281	0.662	0.304	0.700	0.305	0.498	0.101	0.358	0.160	0.827
Gender (G)	0.457	<.0001	0.009	0.393	0.006	0.264	0.354	0.052	0.556	0.629	0.011	0.294	0.123	0.019	0.002
L x G	0.485	0.810	0.254	0.539	0.518	0.924	0.543	0.744	0.815	0.053	0.157	0.275	0.939	0.006	0.179
Block	0.013	0.002	0.238	0.644	0.514	0.111	<.0001	0.0009	0.002	0.084	0.004	0.001	0.073	0.014	0.010

**Table 8:** Effects of providing different colored LED lights during incubation on relative spleen weight (g kg^−1^) of broiler chickens on d 10 of age in the 2nd, 3rd, and 4th trials.

Light	n^1^	Male	Female	Average
Dark	5	0.70 ± 0.033	0.73 ± 0.033	0.72 ± 0.024^ab^
White	6	0.68 ± 0.033	0.66 ± 0.033	0.67 ± 0.024^ab^
Red	6	0.78 ± 0.033	0.70 ± 0.033	0.74 ± 0.024^a^
Blue	6	0.65 ± 0.033	0.64 ± 0.033	0.65 ± 0.024^b^

Average		0.70 ± 0.017	0.68 ± 0.017	

1 Number of experimental units. Experimental unit = 3 pens of birds hatched from the same incubator in trial 2, 3 and 4. ^a,b^Means within a column with different letters differ significantly according to Tukey-Kramer test (α = 0.05).

Male birds hatched under red light had heavier (*P* < 0.05) relative bursa of Fabricius weight than male birds hatched under blue light on d 35 of age (Table 9). No differences (*P* > 0.05) in relative bursa of Fabricius weight were found among light color treatments in female broilers. Interestingly, we observed the lower relative bursa of Fabricius weight in day-old male chicks when illuminated with blue light, but no differences in relative bursa of Fabricius weight were found in female birds (Li et al., 2021). Thus far, the effects of incubation lighting on bursa of Fabricius development are still unclear, especially for the difference between males and females. Birds hatched under red light had higher total IgG concentration than blue, white, and dark groups on d 14 of age (Table 10). Having green or blue lights in broiler houses having positive effects on both humoral and cellular immune response have been reported by many studies (Xie et al., 2008a,b; Firouzi et al., 2014; Zhang et al., 2014). However, providing light exposure with short wavelength (in this case, blue light) during incubation did not increase total chicken IgG level postvaccination in our study. Differences in animal age, time of light exposure and immunoglobulin specificity may explain differences observed.

**Table 9:** Effects of providing different colored LED lights during incubation on relative bursa of Fabricius weight (g kg^−1^) of broiler chickens on d 35 of age in the 2nd, 3rd, and 4th trials.

Light	n^1^	Male	Female
Dark	5	1.69 ± 0.093^ab^	1.32 ± 0.093^b^
White	6	1.66 ± 0.084^ab^	1.51 ± 0.084^ab^
Red	6	1.74 ± 0.084^a^	1.44 ± 0.084^ab^
Blue	6	1.30 ± 0.084^b^	1.53 ± 0.084^ab^

Average		1.60 ± 0.043^x^	1.45 ± 0.043^y^

1 Number of experimental units. Experimental unit = 3 pens of birds hatched from the same incubator in trial 2, 3, and 4. ^a,b^Means within a column with different letters differ significantly according to Tukey-Kramer test (α = 0.05). ^x,y^Means within a row with different letters differ significantly according to Tukey-Kramer test (α = 0.05).

**Table 10:** Effects of providing different colored LED lights during incubation on serum IgG concentration (ng mL^−1^) of broiler chickens on d 14 of age in the 2nd, 3rd, and 4th trials.

Light	n^1^	Serum IgG concentration
Dark	5	547,898 ± 33,381^b^
White	6	601,279 ± 29,660^b^
Red	6	721,158 ± 29,660^a^
Blue	6	570,200 ± 29,660^b^

Effect	ANOVA *P*-value	

Lighting (L)	0.0015	
Gender (G)	0.2871	
L x G	0.0887	
Block	0.0115	

1 Number of experimental units. Experimental unit = 3 pens of birds hatched from the same incubator in trial 2, 3 and 4. ^a,b,c^Means within a column with different letters differ significantly according to Tukey-Kramer test (α = 0.05)

In conclusion, our study shows that providing photoperiodic LED light illumination with different colors did not affect production or health parameters of broilers at market age, but we did find that in ovo photostimulation with blue or white light resulted in an increased feed consumption and body weight, and smaller difference in cloaca temperatures between morning and evening for chicks at a young age, which gives birds a better start for early posthatch development. Furthermore, red light may alter humoral immune response to Newcastle-bronchitis vaccine on d 14 of age. The effects of illumination with different colors during incubation on bird performance can provide useful information for hatchery managers when they practice posthatch feeding programs within the incubator.

## DISCLOSURES

The authors declare that they have no known competing financial interests or personal relationships that could have appeared to influence the work reported in this paper.

## Appendix Supplementary materials

Image, application 1

## Footnotes

Supplementary material associated with this article can be found, in the online version, at doi:10.1016/j.psj.2021.101328.

## References

[bib0001] Archer G.S. (2017). Exposing broiler eggs to green, red and white light during incubation. Animal.

[bib0002] Archer G.S., Jeffrey D., Tucker Z. (2017). Effect of the combination of white and red LED lighting during incubation on layer, broiler, and Pekin duck hatchability. Poult. Sci..

[bib0003] Archer G.S., Shivaprasad H.L., Mench J.A. (2009). Effect of providing light during incubation on the health, productivity, and behavior of broiler chickens. Poult. Sci..

[bib0004] Archer G.S., Mench J.A. (2013). The effects of light stimulation during incubation on indicators of stress susceptibility in broilers. Poult. Sci..

[bib0005] Archer G.S., Mench J.A. (2014). The effects of the duration and onset of light stimulation during incubation on the behavior, plasma melatonin levels, and productivity of broiler chickens. J. Anim. Sci..

[bib0006] Archer G.S., Mench J.A. (2014). Natural incubation patterns and the effects of exposing eggs to light at various times during incubation on post-hatch fear and stress responses in broiler (meat) chickens. Appl. Anim. Behav. Sci..

[bib0007] Archer G.S., Mench J.A. (2017). Exposing avian embryos to light affects post-hatch anti-predator fear responses. Appl. Anim. Behav. Sci..

[bib0008] Balážová L., Baranyiová E. (2010). Broiler response to open field test in early ontogeny. Acta Vet. Brno..

[bib0073] Canadian Council of Animal Care (2009). Guidelines on: The care and use of farm animals in research, teaching and testing.

[bib0009] Classen H.L., Riddell C. (1989). Photoperiodic effects on performance and leg abnormalities in broiler chickens. Poult. Sci..

[bib0010] Di Rosa V., Frigato E., López-Olmeda J.F., Sánchez-Vázquez F.J., Bertolucci C. (2015). The light wavelength affects the ontogeny of clock gene expression and activity rhythms in zebrafish larvae. PLoS One.

[bib0011] Dishon L., Avital-Coehen N., Malamud D., Heiblum R., Druyan S., Porter T.E., Gumulka M., Rozenboim I. (2017). In-ovo monochromatic light photostimulation enhances embryonic somatotropic axis activity. Poult. Sci..

[bib0012] Espinar A., Osuna C., Feliu C., Guerrero J.M. (1994). High activity of retinal N-acetyltransferase in the early development of the chick embryo: independence of lighting conditions. Neurosci. Lett..

[bib0013] Fehrer S.C., Silsby J.L., Burke W.H., El Halawani M.E. (1985). The influence of pharmacological manipulation of serotonin on serum growth hormone and luteinizing hormone levels in young turkeys (Meleagris gallopavo). Poult. Sci..

[bib0014] Firouzi S., Nazarpak H.H., Habibi H., Jalali S.S., Nabizadeh Y., Rezaee F., Ardali R., Marzban M. (2014). Effects of color lights on performance, immune response and hematological indices of broilers. J. World's Poult. Res..

[bib0015] Furuse M., Tachibana T., Ohgushi A., Ando R., Yoshimatsu T., Denbow D.M. (2001). Intracerebroventricular injection of ghrelin and growth hormone releasing factor inhibits food intake in neonatal chicks. Neurosci. Lett..

[bib0016] Gross W.B., Siegel H.S. (1983). Evaluation of the heterophil/lymphocyte ratio as a measure of stress in chickens. Avian Dis.

[bib0017] Halevy O., Biran I., Rozenboim I. (1998). Various light source treatments affect body and skeletal muscle growth by affecting skeletal muscle satellite cell proliferation in broilers. Comp. Biochem. Physiol. A.

[bib0018] Halevy O., Piestun Y., Rozenboim I., Yablonka-Reuveni Z. (2006). In ovo exposure to monochromatic green light promotes skeletal muscle cell proliferation and affects myofiber growth in posthatch chicks. Am. J. Physiol. Regul. Integr. Comp. Physiol..

[bib0019] Hanafy A.M., Hegab I.M. (2019). Effects of egg weight and light sources during incubation period on embryonic development and post-hatch growth of Japanese quail (Coturnix japonica). Europ. Poult. Sci..

[bib0020] Havenstein G.B., Ferket P.R., Qureshi M.A. (2003). Growth, livability, and feed conversion of 1957 versus 2001 broilers when fed representative 1957 and 2001 broiler diets. Poult. Sci..

[bib0021] Huth J.C., Archer G.S. (2015). Effects of LED lighting during incubation on layer and broiler hatchability, chick quality, stress susceptibility and post-hatch growth. Poult. Sci..

[bib0022] Isobe Y., Torri T., Konishi E., Fujioi J. (2002). Effects of melatonin injection on running-wheel activity and body temperature differ by the time of day. Pharmacol. Biochem. Behav..

[bib0023] Kaiya H., Furuse M., Miyazato M., Kangawa K. (2009). Current knowledge of the roles of ghrelin in regulating food intake and energy balance in birds. Gen. Comp. Endocrinol..

[bib0024] Klein D.C., Weller J.L. (1972). Rapid Light-Induced Decrease in Pineal Serotonin N-Acetyltransferase Activity. Science.

[bib0025] Kliger C.A., Gehad A.E., Hulet R.M., Roush W.B., Lillehoj H.S., Mashaly M.M. (2000). Effects of photoperiod and melatonin on lymphocyte activities in male broiler chickens. Poult. Sci..

[bib0027] Li J., Cao J., Wang Z., Dong Y., Chen Y. (2015). Melatonin plays a critical role in inducing B lymphocyte proliferation of the bursa of Fabricius in broilers via monochromatic lights. J. Photochem. Photobiol. B, Biol..

[bib0028] Li X., Rathgeber B., McLean N, MacIsaac J. (2021). Providing Colored Photoperiodic Light Stimulation During Incubation: 1. Effects on Embryo Development and Hatching Performance in Broiler Hatching Eggs.

[bib0029] McKeown B.A., John J.M., George J.C. (1975). Diurnal variation of effects of melatonin on plasma growth hormone and glucose in the pigeon. Endocrinol. Exp..

[bib0030] Mishra I., Singh D., Kumar V. (2016). Daily expression of genes coding for neurotransmitters in central and peripheral tissues of redheaded bunting: implication for circadian regulation of physiology in songbirds. Chronobiol. Int..

[bib0031] Murakami N., Kawano T., Nakahara K., Nasu T., Shiota K. (2001). Effect of melatonin on circadian rhythm, locomotor activity and body temperature in the intact house sparrow, Japanese quail and owl. Brain Res.

[bib0032] Nakamura K. (2011). Central circuitries for body temperature regulation and fever. Am. J. Physiol. Regul. Integr. Comp. Physiol..

[bib0033] National Chicken Council. 2020. U.S. broiler performance. [Online] available: https://www.nationalchickencouncil.org/about-the-industry/statistics/u-s-broiler-performance/ [Accessed Sept. 2020].

[bib0034] National Research Council (1994). Nutrient Requirements of Poultry..

[bib0035] Olanrewaju H.A., Thaxton J.P., Dozier W.A., Purswell Joseph, Roush W.B., Branton S.L. (2006). A review of lighting programs for broiler production. Int. J. Poult. Sci..

[bib0036] Özkan S., Yalçın S., Babacanoğlu E., Uysal S., Karadaş F., Kozanoğlu H. (2012). Photoperiodic lighting (16 hours of light: 8 hours of dark) programs during incubation: 2. Effects on early posthatching growth, blood physiology, and production performance in broiler chickens in relation to posthatching lighting programs. Poult. Sci..

[bib0037] Pardue S.L., Thaxton J.P., Brake J. (1985). Role of ascorbic acid in chicks exposed to high environmental temperature. J. Appl. Physiol..

[bib0038] Prescott N.B., Wathes C.M. (1999). Spectral sensitivity of the domestic fowl (Gallus g. domesticus). Br. Poult. Sci..

[bib0039] Quinteiro-Filho W.M., Ribeiro A., Ferraz-de-Paula V., Pinheiro M.L., Sakai M., Sá L.R.M.D., Ferreira A.J.P., Palermo-Neto J. (2010). Heat stress impairs performance parameters, induces intestinal injury, and decreases macrophage activity in broiler chickens. Poult. Sci..

[bib0040] Reppert S.M., Weaver D.R. (2002). Coordination of circadian timing in mammals. Nature.

[bib0041] Riddell C., Classen H.L. (1992). Effects of increasing photoperiod length and anticoccidials on performance and health of roaster chickens. Avian Dis.

[bib0042] Rogers L.J. (2008). Development and function of lateralization in the avian brain. Brain Res. Bull..

[bib0043] Rozenboim I., Biran I., Uni Z., Robinzon B., Halevy O. (1999). The effect of monochromatic light on broiler growth and development. Poult. Sci..

[bib0044] Rozenboim I., Piestun Y., Mobarkey N., Barak M., Hoyzman A., Halevy O. (2004). Monochromatic light stimuli during embryogenesis enhance embryo development and posthatch growth. Poult. Sci..

[bib0045] Rozenboim I., Biran I., Chaiseha Y., Yahav S., Rosenstrauch A., Sklan D., Halevy O. (2004). The effect of a green and blue monochromatic light combination on broiler growth and development. Poult. Sci..

[bib0046] Rozenboim I., Huisinga R., Halevy O., El Halawani M.E. (2003). Effect of embryonic photostimulation on the posthatch growth of turkey poults. Poult. Sci..

[bib0047] Saarela S., Heldmaier G. (1987). Effect of photoperiod and melatonin on cold resistance, thermoregulation and shivering/nonshivering thermogenesis in Japanese quail. J. Comp. Physiol. B.

[bib0048] Sabuncuoğlu K.M., Korkmaz F., Gürcan E.K., Narinç D., Şamlı H.E. (2018). Effects of monochromatic light stimuli during embryogenesis on some performance traits, behavior, and fear responses in Japanese quails. Poult. Sci..

[bib0049] SAS Institute (2013). SAS User's Guide: Statistics. Version 9.4.

[bib0050] Scanes C.G., Radecki S.V., Malamed S. (1992). Mechanisms involved in the avian patterns of growth hormone secretion during growth and development. Ornis Scand.

[bib0051] Schwean-Lardner K., Fancher B.I., Gomis S., Van Kessel A., Dalal S., Classen H.L. (2013). Effect of day length on cause of mortality, leg health, and ocular health in broilers. Poult. Sci..

[bib0052] Shafey T.M., Al-mohsen T.H. (2002). Embryonic growth, hatching time and hatchability performance of meat breeder eggs incubated under continuous green light. Asian-Aust. J. Anim. Sci..

[bib0053] Siegel P.B., Isakson S.T., Coleman F.N., Huffman B.J. (1969). Photoacceleration of development in chick embryos. Comp. Biochem. Physiol..

[bib0054] Sinkalu V.O., Ayo J.O., Adelaiye A.B., Hambolu J.O. (2015). Ameliorative effects of melatonin administration and photoperiods on diurnal fluctuations in cloacal temperature of Marshall broiler chickens during the hot dry season. Int. J. Biometeorol..

[bib0055] Soliman E.S., Hassan R.A. (2019). Impact of lighting color and duration on productive performance and Newcastle disease vaccination efficiency in broiler chickens. Vet. World.

[bib0056] Sultana S., Hassan M.R., Choe H.S., Ryu K.S. (2013). The effect of monochromatic and mixed LED light colour on the behaviour and fear responses of broiler chicken. Avian Biol. Res..

[bib0057] Underwood H., Edmonds K. (1995). The circadian rhythm of thermoregulation in Japanese quail: III. Effects of melatonin administration. J. Biol. Rhythms.

[bib0058] Van der Pol C.W., van Roovert-Reijrink I.A.M., Aalbers G., Kemp B., van den Brand H. (2017). Incubation lighting schedules and their interaction with matched or mismatched post hatch lighting schedules: effects on broiler bone development and leg health at slaughter age. Res. Vet. Sci..

[bib0059] Wabeck C.J., Skoglund W.C. (1974). Influence of radiant energy from fluorescent light sources on growth, mortality, and feed conversion of broilers. Poult. Sci..

[bib0060] Wang T., Wang Z., Cao J., Dong Y., Chen Y. (2014). Monochromatic light affects the development of chick embryo liver via an anti-oxidation pathway involving melatonin and the melatonin receptor Mel1c. Can. J. Anim. Sci..

[bib0061] Wiebe M.O., Evans R.M. (1994). Development of temperature regulation in young birds: evidence for a vocal regulatory mechanism in two species of gulls (Laridae). Can. J. Zool..

[bib0062] Xie D., Wang Z.X., Dong Y.L., Cao J., Wang J.F., Chen J.L., Chen Y.X. (2008). Effects of monochromatic light on immune response of broilers. Poult. Sci..

[bib0063] Xie D., Wang Z., Cao J., Dong Y., Chen Y. (2008). Effects of monochromatic light on proliferation response of splencyte in broilers. Anat. Histol. Embryol..

[bib0064] Yahav S. (2015). Regulation of body temperature: strategies and mechanisms. Sturkie’s Avian Physiology..

[bib0065] Zawilska J.B., Jarmak A., Woldan-Tambor A., Nowak J.Z. (1995). Light-induced suppression of nocturnal serotonin N-acetyltransferase activity in chick pineal gland and retina: A wavelength comparison. J. Pineal Res..

[bib0067] Zeman M., Gwinner E., Herichova I., Lamošová D., Košt'ál L. (1999). Perinatal development of circadian melatonin production in domestic chicks. J. Pineal Res..

[bib0068] Zhang L., Zhang H.J., Qiao X., Yue H.Y., Wu S.G., Yao J.H., Qi G.H. (2012). Effect of monochromatic light stimuli during embryogenesis on muscular growth, chemical composition, and meat quality of breast muscle in male broilers. Poult. Sci..

[bib0069] Zhang Z., Cao J., Wang Z., Dong Y., Chen Y. (2014). Effect of a combination of green and blue monochromatic light on broiler immune response. J. Photochem. Photobiol. B: Biol..

[bib0070] Zhang L., Zhu X.D., Wang X.F., Li J.L., Gao F., Zhou G.H. (2016). Green light-emitting diodes light stimuli during incubation enhances posthatch growth without disrupting normal eye development of broiler embryos and hatchlings. Asian Aust. J. Anim. Sci..

[bib0071] Zhou W.T., Yamamoto S. (1997). Effects of environmental temperature and heat production due to food intake on abdominal temperature, shank skin temperature and respiration rate of broilers. Br. Poult. Sci..

[bib0072] Zulkifli I., Rasedee A., Syaadoh O., Norma M.T. (1998). Daylength effects on stress and fear responses in broiler chickens. Asian Aust. J. Anim. Sci..

