# Supplementary material for: Providing colored photoperiodic light stimulation during incubation: 2. Effects on early posthatch growth, immune response, and production performance in broiler chickens
Source: Poult Sci. 2021 Jun 12;100(9):101328. doi: 10.1016/j.psj.2021.101328 (PMC8318990; doi:10.1016/j.psj.2021.101328)
Supplement: Supplementary file 1 [file mmc1.docx]

Supplementary Table 1. Effect of providing different colored LED lights during incubation on body weight (g bird^-1^) of broiler chickens.

|  |  |  |  |  |  |  | Day |  |  |  |  |
| --- | --- | --- | --- | --- | --- | --- | --- | --- | --- | --- | --- |
| Light | n^1^ | 1 | 2 | 3 | 4 | 5 | 6 | 7 | 14 | 25 | 35 |
| Dark | 7 | 52.8 | 65.9 | 83.7 | 104.2 | 126.0 | 153.0 | 181.3 | 474.4 | 1390.4 | 2523.9 |
| White | 8 | 54.0 | 67.3 | 85.4 | 105.9 | 127.7 | 155.0 | 183.6 | 484.0 | 1394.5 | 2537.2 |
| Red | 8 | 53.4 | 66.5 | 84.3 | 104.8 | 126.5 | 153.5 | 181.0 | 481.1 | 1395.3 | 2539.2 |
| Blue | 8 | 54.0 | 67.2 | 85.1 | 105.3 | 126.8 | 154.1 | 182.5 | 480.6 | 1395.3 | 2535.7 |
| SEM |  | 2.3 | 2.6 | 3.6 | 4.2 | 3.8 | 6.0 | 9.5 | 14.3 | 72.1 | 176.2 |

^1^Number of experimental units. Experimental unit = 3 pens of birds hatched from the same incubator.
